# Prevention of anastomotic leakage using a polyglycolic acid sheet in double-stapling technique anastomosis for rectal surgery

**DOI:** 10.1016/j.amsu.2021.103117

**Published:** 2021-11-24

**Authors:** Masatsune Shibutani, Hisashi Nagahara, Tatsunari Fukuoka, Yasuhito Iseki, Yuki Okazaki, Kosei Hirakawa, Masaichi Ohira

**Affiliations:** Department of Gastroenterological Surgery, Osaka City University Graduate School of Medicine, Osaka, Japan

**Keywords:** Polyglycolic acid, Anastomotic leakage, Rectal surgery, Double-stapling technique, Prevention, Case series

## Abstract

**Background:**

Due to the development of surgical techniques and devices, the incidence of anastomosis leakage in rectal surgery has decreased. However, anastomotic leakage in rectal surgery remains a serious postoperative complication. The present study examined whether or not a polyglycolic acid (PGA) sheet is effective for reinforcing rectal anastomosis.

**Material and methods:**

Fifteen patients who underwent double-stapling technique (DST) anastomosis during rectal surgery were enrolled in this study. The PGA sheet was used as the reinforcing material. DST anastomosis was performed with the PGA sheet sandwiched, and a strip of the PGA sheet was wrapped around the anastomosis.

**Results:**

No patients had anastomotic leakage.

**Conclusion:**

A PGA sheet may be effective for preventing anastomotic leakage in DST anastomosis for rectal surgery.

## Highlights

• The new method for preventing anastomotic leakage in rectal surgery.
• A polyglycolic acid (PGA) sheet which is absorbable reinforcement material was used.
• Thanks to the use of the PGA sheet, no patients had anastomotic leakage.

## 1 Introduction

In rectal surgery, the incidence of anastomotic leakage has been reported to be approximately 10% [[1], [2], [3]]. Anastomotic leakage causes peritonitis, resulting in re-operation, long-term fasting and long-term hospitalization. Furthermore, anastomotic leakage has a negative impact on the long-term survival via the promotion of inflammatory cytokine production [4,5]. Therefore, various surgical techniques have been devised. For example, an evaluation of the intestinal blood flow using indocyanine green (ICG) fluorescent [6,7], sufficient mobilization of the left colon including splenic flexure for tension-free anastomosis [8,9], improvements in stapling devices [10,11] and the placement of a transanal tube for decompression of anastomosis [12,13]. However, anastomotic leakage in rectal surgery remains one of the most serious postoperative complications.

The present study examined whether or not a polyglycolic acid (PGA) sheet, which prevents air leakage after lung surgery and pancreatic fistula after pancreatic surgery [14,15], is effective for reinforcing rectal anastomosis.

## 2 Methods

### 2.1 Patients

Fifteen patients who underwent double-stapling technique (DST) anastomosis during non-consecutive open/laparoscopic rectal surgery for benign/malignant disease at the Osaka City University Hospital from December 2020 to June 2021 were enrolled in this study. Patients who received neoadjuvant therapy or emergency surgery were excluded from this study. All procedures were performed by senior surgeons specialized in colorectal surgery. All patients received a follow-up of 3 months or longer at the Osaka City University Hospital. This retrospective study was approved by the Ethics Committee of Osaka City University (approval number: 4182) and conducted in accordance with the Declaration of Helsinki. All patients provided their written informed consent.

### 2.2 How to attach the reinforcing material to a circular stapler

**Fig. 1:**
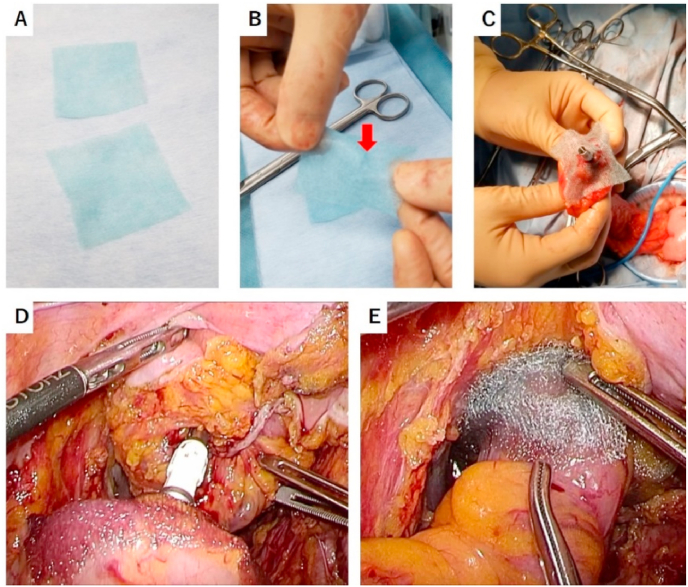
How to attach the reinforcing material to a circular stapler. (A) Cutting, (B) making a slit, (C) attachment to the anvil, (D) anastomosis with the polyglycolic acid sheet sandwiched, (E) wrapping.

The PGA sheet (Neoveil®; Gunze, Kyoto, Japan) was used as the reinforcing material. First, the PGA sheet was cut in half (Fig. 1A). Second, a slit of a few millimeters was made in the center of the sheet (Fig. 1B) and attached to the anvil (Fig. 1C). Third, DST anastomosis was performed with the PGA sheet sandwiched (Fig. 1D). Finally, a strip of the PGA sheet was wrapped around the anastomosis (Fig. 1E).

This case series has been reported in line with the PROCESS Guideline [16], and has been registered with a Research Registry (UIN: 7387, http://www. researchregistry.com/on November 21, 2021).

## 3 Results

Details regarding the surgical procedure are shown in Table 1. The distribution of operation methods are as follows: high-anterior resection, 10; low-anterior resection, 5 patients. No patients had anastomotic leakage, anastomotic stenosis or postoperative complications due to intrapelvic adhesion, such as ileus.

**Table 1:** Patient characteristics and details regarding the surgical procedure.

Age (years)	
Median (range)	69 (51–87)
Gender	
Male	11
Female	4
Operation methods	
High anterior resection	10
Low anterior resection	5
Number of stapler cartridges for rectal transection	
1	10
2	5
Circular Stapler	
TRI-EEA28	10
ECS25	2
Powered ECS25	2
ECS29	1

Abbreviations: TRI-EEA = EEA stapler with Tri-staple, ECS = Echelon circular stapler.

## 4 Discussion

We determined the efficacy of intracorporeal reinforcing sutures for preventing anastomotic leakage after rectal surgery [17]. However, in our previous study, the incidence of anastomotic leakage was 5.6%, indicating room for improvement with regard to rectal anastomosis [17]. In the present study, thanks to the use of the PGA sheet, no patients had anastomotic leakage, although the study included only a small number of cases.

The PGA sheet used in this study is an absorbable reinforcement material that is easy to use anywhere in the body due to its thin and soft features. The PGA sheet has been reported to be more effective for preventing the postoperative complications than other reinforcing materials and has been widely used for various organs based on substantial evidence [14,15,18,19]. The PGA sheet has been reported to be effective in preventing air leakage after lung surgery and pancreatic fistula after pancreatic surgery by forming a barrier due to thickening of the collagen tissue associated with inflammation within a few days after surgery [14,15]. Furthermore, in an experimental study using fresh porcine small intestine, it was also reported that the PGA sheet contributed to the stabilization of staples by sealing off the staple holes and acting as a neutralization plate, resulting in an increase in pressure resistance [20,21].

**Fig. 2:**
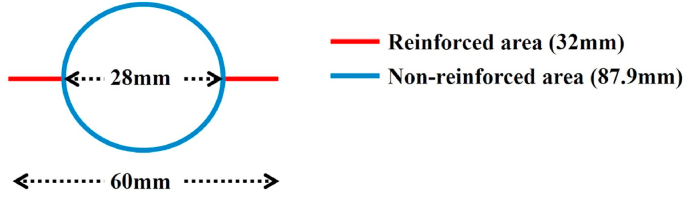
When performing DST anastomosis in the center of the staple line of a linear stapler (60 mm) with a circular stapler (28 mm), only about 30% of the total staple line is reinforced. Red line: 32.0 mm, blue line: 87.9 mm. (For interpretation of the references to color in this figure legend, the reader is referred to the Web version of this article.)

**Fig. 3:**
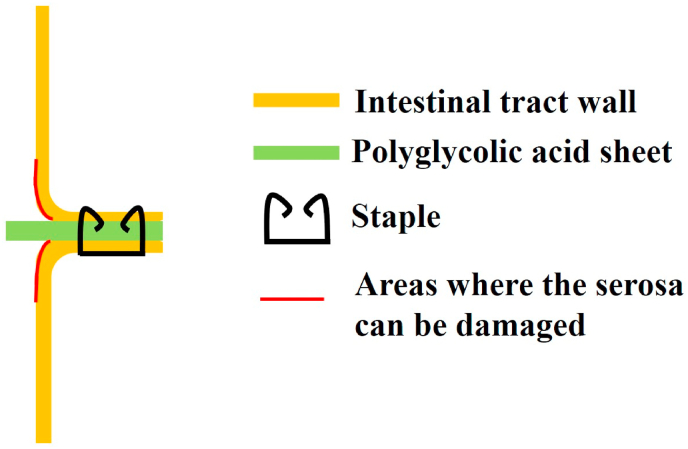
Areas where small fissures may occur on the serosal surface.

Sutures are means of reinforcing anastomosis. However, suturing the posterior wall is often difficult, although suturing the front wall and the crossing point of the staple lines is often possible. Furthermore, in cases of low level of anastomosis, suturing itself is difficult. Even when using a stapling device that combines the PGA sheet and linear stapler, circular staple lines cannot be reinforced, so the staple line reinforcement rate is only about 30% (Fig. 2). In contrast, DST anastomosis using the PGA sheet is very easy to perform and does not require training. In addition, this procedure can be used for low level of anastomosis and can reinforce the entire range of the staple line. The wrapping method, which has been reported to be able to increase the strength of vessel walls [18], may also be effective for small fissures that may occur on the serosal surface caused by tightening during anastomosis or detachment of the mesentery (Fig. 3). Furthermore, a PGA sheet is not very expensive. However, one reported disadvantage of PGA is that adhesions can occur between the thoracic wall and lung when using a PGA sheet for pleural defect repair in lung surgery [14]. Of note, no ileus due to adhesion of the intestine around the DST anastomosis developed in the present study.

Several limitations associated with the present study should be mentioned. First, the current study was a retrospective study of a small cohort in a single center. Second, there is some evidence of histological changes caused by PGA with respect to the lung and pancreas, but there has been no evidence of histological changes with respect to intestinal tract. The only evidence suggesting reinforcement of the intestinal tract was the increase in PGA-induced physical pressure resistance. Third, the influence of the wrapping method on the intestinal tract is unclear, although there is some evidence suggesting a reinforcing effect of the wrapping method on blood vessels [18]. Fourth, we did not examine whether applying the PGA sheet to the serosal side or the mucosal side was more effective in reinforcing the anastomosis.

Randomized controlled trials will be needed to verify the efficacy of the PGA sheet in DST anastomosis for rectal surgery. The effect of the PGA sheet on preventing anastomotic leakage may not only help reduce the incidence of anastomotic leakage but also avoid the need for diverting stoma.

## 5 Conclusion

The PGA sheet may be effective for preventing anastomotic leakage in DST anastomosis for rectal surgery.

## Ethical approval

This retrospective study was approved by the Ethics Committee of Osaka City University (approval number: 4182) and conducted in accordance with the Declaration of Helsinki.

## Sources of funding

The authors declare that they have no funding source for this article.

## Author contribution

Masatsune Shibutani: study conception, design, critical revision of the manuscript and drafting of the manuscript.

Hisashi Nagahara, Tatsunari Fukuoka, Yasuhito Iseki, Yuki Okazaki, Kosei Hirakawa and Masaichi Ohira: critical revision of the manuscript.

All authors contributed to the article and approved the submitted version.

## Consent

All patients provided their written informed consent.

## Registration of research studies

1. Name of the registry: Research Registry
2. Unique Identifying number or registration ID: 7387
3. Hyperlink to your specific registration (must be publicly accessible and will be checked): http://www.researchregistry.com

## Guarantor

Masatsune Shibutani.

## Provenance and peer review

Not commissioned, externally peer-reviewed.

## Declaration of competing interest

The authors declare that they have no conflict of interest for this article.
